# The Potential of Algae in the Nutricosmetic Sector

**DOI:** 10.3390/molecules28104032

**Published:** 2023-05-11

**Authors:** Irene Dini

**Affiliations:** Department of Pharmacy, University of Naples Federico II, Via Domenico Montesano 49, 80131 Napoli, Italy; irdini@unina.it

**Keywords:** food supplement, nutraceutical, seaweed, circular economy, biodiversity recycling, eco-friendly product, waste management, peptides, antioxidants, phenolics

## Abstract

Seaweeds or algae are marine autotrophic organisms. They produce nutrients (e.g., proteins, carbohydrates, etc.) essential for the survival of living organisms as they participate in biochemical processes and non-nutritive molecules (such as dietary fibers and secondary metabolites), which can improve their physiological functions. Seaweed polysaccharides, fatty acids, peptides, terpenoids, pigments, and polyphenols have biological properties that can be used to develop food supplements and nutricosmetic products as they can act as antibacterial, antiviral, antioxidant, and anti-inflammatory compounds. This review examines the (primary and secondary) metabolites produced by algae, the most recent evidence of their effect on human health conditions, with particular attention to what concerns the skin and hair’s well-being. It also evaluates the industrial potential of recovering these metabolites from biomass produced by algae used to clean wastewater. The results demonstrate that algae can be considered a natural source of bioactive molecules for well-being formulations. The primary and secondary metabolites’ upcycling can be an exciting opportunity to safeguard the planet (promoting a circular economy) and, at the same time, obtain low-cost bioactive molecules for the food, cosmetic, and pharmaceutical industries from low-cost, raw, and renewable materials. Today’s lack of methodologies for recovering bioactive molecules in large-scale processes limits practical realization.

## 1. Introduction

The main goal of the Circular Economy is to reuse and recycle natural resources to minimize health, energy, and environmental impacts. The European citizen produces around 5 tonnes of waste, much of which finishes in incinerators or landfills, and a little is recycled [1]. Waste management policies have been investigated to avoid landfills and allow the recovery of renewable energy and recycled materials [2]. Organizations have developed circular waste management systems, promoting resource flow and enhancing product sustainability and processes [3]. Consumption of eco-friendly products and decreasing waste are crucial to achieving the European sustainable goals. Ten megatrends were recognized for 2022 by New Nutrition Business for food, nutrition, and health. Sustainability came fifth [4]. Representative population surveys indicate that many people (amongst them young consumers) wish to contribute to sustainable development [5,6,7,8,9,10]. Buying eco-friendly products is considered one way to intervene. In the European Union, 26% of consumers purchase eco-friendly products, and 54% rarely use such items [11]. The global market value of natural and organic skincare products will probably grow from 9.9 billion dollars in 2021 to 20.4 billion dollars by 2030 [12]. The organic segment (made from plant ingredients that have been grown in soil free of fungicides, pesticides, synthetic fertilizers, and herbicides, and genetically modified organisms) was valued at $28,323.2 million in 2021 and is expected to reach $74,058.5 million by (CAGR of 9.8%) [13]. This data supports the significant contribution of the cosmetics market worldwide to environmental sustainability. The seaweed waste (e.g., beach-casts) [14] and invasive species valorization [15], which are of no commercial value and must be disposed of in landfills, could represent an eco-friendly, attractive low-cost source for supplements and cosmetics formulations. Some scientific studies have shown the potential skincare properties of algae bioactive metabolites [16,17,18,19]. In seaweeds are found compounds with low allergen and cytotoxic profiles [20], such as peptides, polysaccharides, fatty acids, vitamins, carotenoids, phlorotannins, tocopherols, phycobilins, phycocyanins, and sterols [21,22,23,24] that can act as antioxidants, photoprotective, moisturizing, anti-inflammatory, antiallergic, anti-acne, anti-wrinkling, antiaging, antimicrobial, and whitening bioactive compounds [25,26,27,28]. The present review summarizes the algal functional and technological properties to highlight their use for the nutricosmetic market and provide reasons for reflection for subsequent studies. Bibliometric works published between 1991 and 2023 collected in two central citation databases (Scopus and Web of Science) were consulted for the work’s drafting.

## 2. Nutricosmetic Revolution

The term “nutricosmetic” indicates the association of food supplements and cosmeceuticals to improve skin care. Nutricosmetic formulations optimize the intake of nutritional macro and micro elements to meet the demands of the skin and appendages, improving their conditions and delaying aging [29,30,31]. A food supplement is a consumer product that aims to supplement the regular diet. Products based on vitamins, minerals, antioxidants, and extracts of vegetable origin, single and multi-compound, in pre-dosed forms with nutritional power or biological effect, fall into the vast category of food supplements [32]. Cosmetics represent a highly heterogeneous category of daily-use consumer products. In the European Union, Regulation (EC) no. 1223/2009 in Article 2 defines “cosmetic product” as “any substance or mixture intended to be applied on the external surfaces of the human body (epidermis, hair system, and hair, nails, lips, external genital organs) or the teeth and on the mucous membranes of the mouth for the sole or primary purpose of cleaning them, perfuming them, modifying their appearance, protecting them, keeping them in good condition or correcting body odors” [33]. A substance or mixture intended to be ingested, inhaled, injected, or implanted in the human body is not considered a cosmetic product. Nutricosmetic formulations combine the two previous formulations’ beneficial effects through an integrated “in and out” approach.

## 3. Algae (Seaweeds)

**Figure 1:**
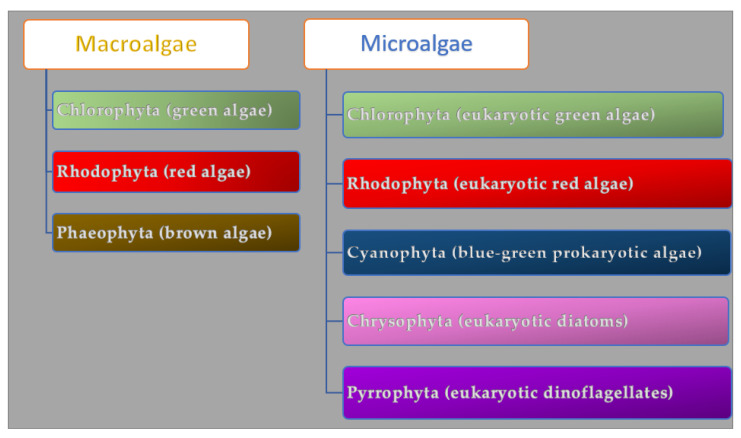
Algae classification.

Algae are a group of photosynthetic organisms that differ in structure and size. They can grow in freshwater, marine water, deep oceans, and rocky shores. The bionetwork comprises 36,000 different kinds of algae. The seaweed macroalgae are multicellular organisms rich in lipids and proteins (40% and 71% of their dry weight) that can measure from a few centimeters to a meter, while the microalgae are microscopic unicellular carbohydrate-rich organisms [34]. Macroalgae are grouped in Chlorophyta (green algae), Phaeophyta (brown algae), and Rhodophyta (red algae) according to their pigment and chlorophyll profile (Figure 1).

Microalgae are classified as prokaryotic and eukaryotic and, according to their color, subdivided into Cyanophyta (blue-green prokaryotic algae), Chlorophyta (eukaryotic green algae), Rhodophyta (eukaryotic red algae), Chrysophyta (golden eukaryotic diatoms), and Pyrrophyta (brown eukaryotic dinoflagellates) [35]. The chlorophyll responsible for the green color of the algae *U. lactuca*, *C. vulgaris* is employed as an antioxidant bioactive compound in cosmetics. Beta-carotene found in *D. salina* [36] and the red protein phycoerythrin found in red algae (e.g., *Porphyra*, *Gracilaria*, *Irish moss*) [37] are used as colorants in foods and cosmetics. The fucoxanthin in brown algae (*Laminaria digitata*, *Isochrysis* spp., *Postelsia palmaeformis*) prevents skin aging (by supporting collagen production and moisturizing skin) and has anti-inflammatory and tyrosinase inhibitory effects [38]. The algae metabolites’ composition is associated with internal factors (i.e., type and species), external factors (i.e., water temperature, water composition, salinity gradient, time of year, organism age), and cultivation conditions such as size and type of cultivation reactor [39]. During stress conditions, algae produce organic phenolic and phlorotannin and improve the uptake of inorganic ions to protect them from UV lights and desiccation. [40]. The wave exposure, environmental gradients, and algae reproductive cycles affect carbohydrate profile and content [41]. Chemicals (e.g., pH, carbon dioxide, salinity mixing/aeration), physical parameters (e.g., light, radiation, temperature), carbon sources (e.g., organic carbon like sugars and CO_2_), nitrogen, salts, phosphorous, and vitamins affect the algaes’ growth [42]. Microalgae can be grown autotrophically, heterotrophically, and mixotrophically. Cellular self-shading and low light availability negatively affect biomass production during autotrophic nutrition. Inorganic carbon sources can enhance biomass concentration and photosynthetic activities [42]. Organic substrates such as sugars, organic acids, etc. (heterotrophic nutrition), give rapid growth, low harvesting costs, and high biomass production [43]. The high cost of organic carbon sources, substrate inhibition, contamination, and the low number of microalgal species that can be grown in this way limit heterotrophic nutrition [44]. Mixotrophic algae can photosynthesize, assimilate, and metabolize organic carbon and are less dependent on light penetration for higher cell densities than autotrophy ones. During dark respiration, they manage biomass decrease, using lower organic substrate amounts than heterotrophic growth and enhancing the synthesis of the PUFA (polyunsaturated fatty acids) [44,45,46]. Algae can improve air quality by fixing CO_2_ [47] (they are responsible for 50% of the photosynthesis on earth) [48] and are an alternative source of bioenergy production since they produce biofuels [49]. Finally, they can reduce pollution [50] by converting water and CO_2_ into organic matter [51].

## 4. Algae Metabolites

### 4.1. Polysaccharides

**Figure 2:**
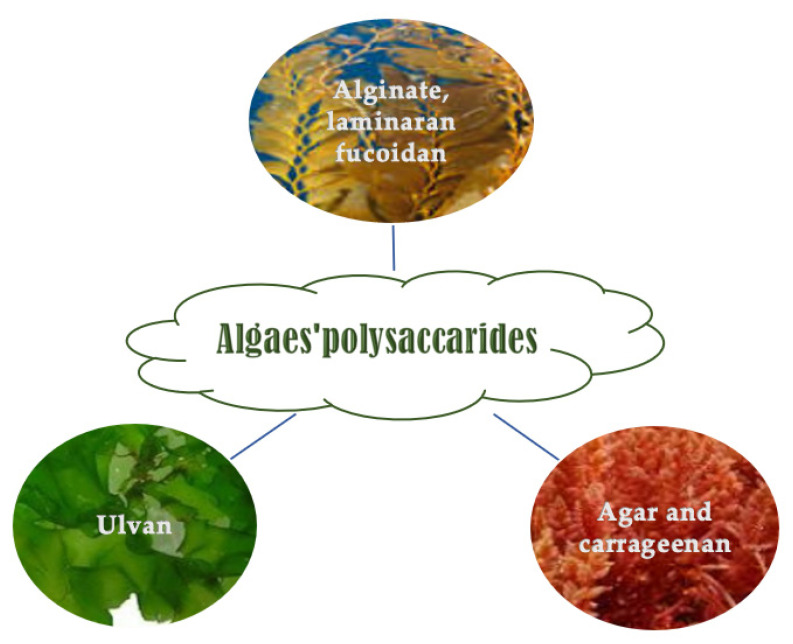
Polysaccharides occurrence in the function of algae class.

Marine macroalgae are good carbohydrate sources (mainly polysaccharides and low concentration of disaccharides and monosaccharides) whose content is from 5 to 75% (*w*/*w*, DW) based on the age, period, species, and harvesting site [52,53]. Polysaccharides in seaweeds can be sulfated and non-sulfated [54]. They constitute the algae cell walls and are species-specific (Figure 2) [55,56]. They have some technological, rheological, and biological activities. They can have a prebiotic effect and improve gut human microbiota performance [57].

#### 4.1.1. Brown Algae Polysaccharides

Brown macroalgae are composed of sulfated and branched α-l-fucans containing predominantly sulfated l-Fuc*p* (<90%), other monosaccharides (e.g., d-Man*p*, d-Gal*p*, and d-Xyl*p)*, and uronic acids (d-GlcA*p* and d-GalA*p*). Brown algae polysaccharides have antioxidant, antiinflammatory, and antibacterial activity against *E. coli*, *S. epidermidis*, *S. aureus*, and *B. licheniformis* [58,59].

Ascophyllans (xylofucoglucuronanes) have a poly-(1→4)-β-d-glucuronan skeleton linked to l-Fuc*p* and d-Xyl*p* sulfated in position C-4 [60].

Sargassans (glucuronofucogalactans), identified in the genus *Sargassum* (e.g., *Sargassum linifolium*), have a poly-(1→4)-β-d-glucuronan skeleton linked with d-Man*p* residues [61].

Fucoidans have low shear-thinning performance and low viscoelastic physical characteristics (they are affected by monovalent and divalent salts) [62]. They are biocompatible, non-toxic, biodegradable [63,64], and have antioxidant and antiradical properties [65,66,67,68]. Fucoidans can promote skin firmness, elasticity, brightness, hair growth, safety, cleanliness, rigidity, and gloss [69]. They prevent and treat skin photoaging, decreasing wrinkle-related enzymes (e.g., collagenase, gelatinase, elastase) [70,71,72], improving collagen synthesis [73], controlling matrix metalloproteinases and avoiding the extracellular matrix’s ruin [74,75,76,77].

Laminarins (also laminarans), identified in *laminaria* present in the North Atlantic, have a degree of polymerization of 15–40 and molecular weight (M_w_ of 2–10 kDa). They are β-(1→3)-d-glucans. The laminaribiosis are the diholosidic repeating unit consisting of β-(1→6)-d-Glc*p* [78]. Laminarins are biocompatible, have low cell toxicity, are biodegradable, and show some bioactivity, such as anti-inflammatory, antioxidant [79] anti-photoaging and regenerative abilities [80].

Alginate(s) are polysaccharides composed of α-l-guluronic acid (l-Gul*p*A) (^1^C_4_ ring conformation) and (1→4)-β-d-mannuronic acid (d-Man*p*A) (^4^C_1_ ring conformation) [81] arranged in both homogeneous and heterogeneous blocks [81]. Alginates are used in the food, feed, cosmetic, and drug industries as gelifying and thickening agents, and bioactive molecules against allergy [82] and obesity [83,84].

#### 4.1.2. Red Algae Polysaccharides

Red algae (Rhodophyta) contain water-soluble sulfated galactan (e.g., agarocolloids and carrageenans), constructed based on (1→4)-α-Gal*p* and (1→3)-β-Gal*p* units [53]. Carrageenans have gel and texture properties. They are the fourth principal hydrocolloids used by the food industry, after starch, gelatin, and pectin [85].

Sulfated dioside are linear polymers of carrabiosis that can contain 4-α-d-Gal*p* and 3-β-d- Gal*p*, other monosaccharides (Xyl*p*, GlcA*p*, Glc*p*, and GalA*p*), methyl ether groups, and pyruvic acid ketals. They are extracted from *Agardhiella*, *Eucheuma*, *Chondrus*, *Gigartina*, *Furcellaria*, and *Hypnea* [53,86].

Agarans are sulfated galactan containing 4-α-l-Gal*p* [87]. Agarans based on the percentages of 3-6-α-l-AnGal*p* residues and sulfate groups are defined agaroids that are weak gelling molecules (divided into funorans and porphyrans), and agars (high gelling molecules). Agaroids are extracted from *Porphyra* species, e.g., *P. capensis*, *Porphyra haitanensis* [88], or *P. umbilicalis* [89]. Agar has cosmetic and pharmaceutical applications as a thickener agent and an ingredient to carry and release drugs in capsules and tablets [89,90].

#### 4.1.3. Green Seaweed Polysaccharides

Chlorophyceae contain sulfated polyholosides [91]. Polyholosides are distinct in sulfated xylorhamnoglycuronans, called ulvans [92,93,94,95], sulfated arabinoxylogalactans or xyloarabinogalactans (composed of Ara*f*, d-Gal*p*, l- and d-Xyl*p* units) present in the orders of Cladophorales and Bryopsidales, and sulfated rhamnogalactogalacturonanes or glucuronoxylorhamnogalactans extracted from *Ulvales* [96]. Ulvans are used as gelling [97] and antiaging agents [98].

### 4.2. Lipids

Algae contain omega-3 and omega-6 polyunsaturated fatty acids (PUFA; usually under 5%). The γ-linolenic acid, eicosapentaenoic acid, arachidonic acid, and docosahexaenoic acid are the most abundant.

*Phaeophyta *algae have a C18-PUFAs profile next to green algae and a C20-PUFAs profile identical to red algae. *Chlorophyta* species have higher levels of C18-PUFAs than C20-PUFAs. In *Rhodophyta* happen the contrary. Green algae contain higher DHA (docosahexaenoic acid) levels (e.g., Chlorophyta algae genus Tetraselmis). Finally, red and brown algae have predominantly EPA (eicosapentaenoic acid), arachidonic acid [99,100], and phospholipids [101,102,103,104,105]. Polyunsaturated fatty acids can improve skin barrier protection [106,107] and regulate inflammatory responses [108]. Lipids in cosmetic formulations can act as moisturizing agents (forming a waterproof film on the skin to avoid water evaporation from the surface) [109], emollient [110], and softening agents (they make the corneocyte’s edges smoother) [36], surfactants [111], and emulsifiers (they decrease the surface tension) [112], texturizers (they improve the spreadability of gel-like products), and as color and fragrance carriers [113].

### 4.3. Proteins and Derivatives

Seaweeds are a rich source of proteins (in single or conjugate form) and protein derivatives (e.g., free amino acids and peptides) [23]. Red algae have the highest proteins and derivative contents (up to 47%), green algae have medium levels (between 9–26%), and brown algae contain the lowest concentrations (3–15%) [114]. Protein and bioactive peptides have high antioxidant, anti-inflammatory, skin proactive, and antiaging properties [115,116,117]. Pedoclimatic conditions affect the proteins, peptides, and amino acids contents in algae.

Taurine extracted from the thalli of *Euthora cristata*, *Ahnfeltia plicata*, and *Ceramium virgatum* has antioxidant and chelating abilities [118,119]. The peptides (PYP1-5, and Porphyra 334) from *Porphyra yezoensis* f. *coreana* increase collagen and elastin levels and reduce the expression of matrix metalloproteinases (MMP) MMP-1 and MMP-8 [120].

**Figure 3:**
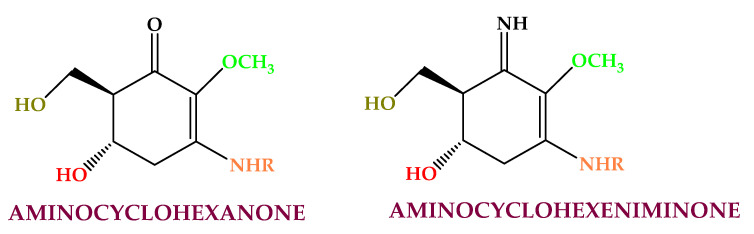
Algaes’ mycosporine-like amino acids found in algae.

Mycosporine-like amino acids (MAAs) (Figure 3) are secondary metabolites with low molecular weight (<400 Da) synthesized for protection against solar radiation and found in the cell cytoplasm [121]. Mycosporine-like amino acids are made by cycloheximide or cyclohexenone conjugated to amino acid or an imino alcohol residue [122]. They are extracted mainly from Rhodophyceae (e.g., shinorine, asterina, porphyra, palythine, polyphenol, mycosporine-glycine, and palythene) [123,124] and from *Asparagopsis armata*, *Mastocarpus stellatus*, *Chondrus crispus*, *Gelidium* sp., *Palmaria palmata*, *Gracilaria cornea*, *Grateloupia lanceola*, *Solieria chordalis*, and *Curdiea racovitzae*. This compound class has shown antioxidant, photoprotective, anti-proliferative [125], anti-aging, and anti-inflammatory activities [126].

MAAs are employed as UV protectors, moisturizing, antiwrinkle, anti-roughness, and cell proliferation stimulators in personal care products and cosmetics [127,128,129].

### 4.4. Phenolics

**Figure 4:**
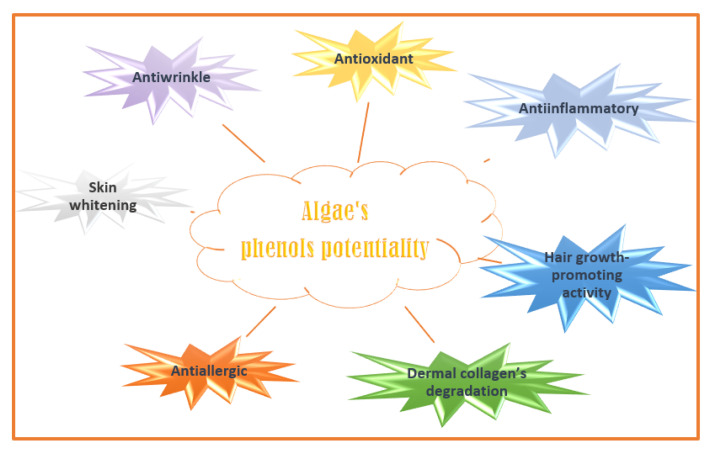
Algaes’ phenols potentialities in nutricosmetic formulation.

Phenolic compounds are secondary plant metabolites with one or more aromatic rings with one or more -OH phenolic groups (e.g., phlorotannins, bromophenols, flavonoids, phenolic terpenoid, and mycosporine-like amino acids) [130]. They can defend algae from pedoclimatic injuries and parasite attacks [131,132]. The biological activities attributed to the algae’s phenolic compounds are summarized in Figure 4 [133].

**Figure 5:**
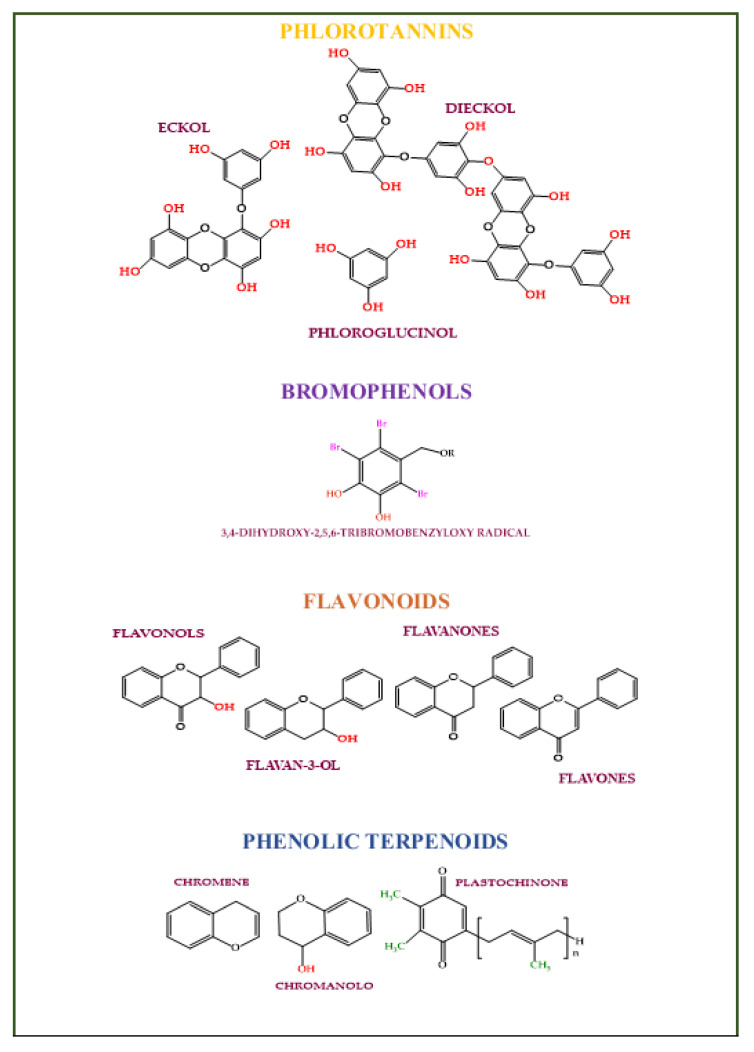
The main class of phenolic compounds found in algae.

Phlorotannins (Figure 5) are phloroglucinol (1,3,5-trihydroxybenzene) polymerized derivatives with ether, phenyl, or 1,4-dibenzodioxin linkages [134,135]. They are found only in brown algae [136]. Phlorotannins have antioxidant activity [137,138,139,140], reduce melanin synthesis, tyrosinase activity [141,142], damages caused by UV rays [143,144], and have anti-inflammatory [145,146], anti-proliferative [147,148,149,150,151], and anti-adipogenic activities [152]. Phlorotannins antioxidant power is 2 to 10 times higher than tocopherol and ascorbic acid [153,154]. Dieckol, eckol, dioxinodehydroeckol, phlorofucofuroeckol A, eckstolonol, and 7-phloroeckol, and decreasing tyrosinase and hyaluronidase activities can act as whiteners and antiwrinkle bioactive compounds in cosmetic formulations [155,156,157,158,159,160]. 7-derived phloroeckol promotes hair growth [161].

Phlorotannins from *Ecklonia kurome* (Phaeophyceae) act as antimicrobial agents against some methicillin-resistant food-borne pathogenic bacteria (*Staphylococcus aureus* strains, *Campylobacter* sp., and *Streptococcus pyogenes*) [162,163].

Dioxinodehydroeckol from *Ecklonia cava* and fucofuroeckol-A derived from the brown seaweed *Ecklonia stolonifera Okamura* can protect against UVB radiation [164,165].

Dieckol from *Ecklonia stolonifera* and other phlorotannins have antiallergic properties [166,167,168]. Phlorotannins also decrease the expression of the interstitial collagenase MMP-1 that regulates the dermal collagen’s degradation in the human skin aging process [169].

Bromophenols (B.P.s) (Figure 4) have one or several benzene rings with bromine and hydroxyl-substituents. They were isolated from red, green, and brown algae [170]. Bromophenols can act as antioxidants [171,172,173,174,175,176], antimicrobials (against *Candida albicans *[177,178], *Pseudomonas fluorescence*, and *Staphylococcus aureus*) [179], anti-inflammatories (decreasing the IgE-mediated responses, the interleukin-6, nuclear factor kappa-light-chain-enhancer, and activator of transcription1 pathways) [180], whitening (inhibiting the tyrosinase enzyme levels) [181], antiobesity, anticancer, and antiosteoporosis bioactive compounds (decreasing carbonic anhydrase [170,182], and glucose 6-phosphate dehydrogenase activities) [183,184].

Flavonoids are molecules derived from the phenylpropanoid metabolism and shikimate pathway. They have a high reduction potential and scavenging activity [185]. Flavones (e.g., luteolin, apigenin, chrysin, and baicalein) were isolated in the *Ulva intestinalis* and *Cladophora vagabunda* green seaweeds [186] and *Phaeocystis globosa* red alga [187]. Catechins (e.g., epicatechin and epigallocatechin) were detected in the *U. pinnatifida* brown seaweeds [188]. Flavonols (e.g., rutin, quercitin) in *Chlorophyta*, *Rhodophyta*, and P*haeophyceae* species [136]. Isoflavones (e.g., daidzein or genistein) are present in red macroalgae (*Chondrus crispus* and *Porphyra*/*Pyropia* spp.) and brown seaweeds (*Sargassum muticum* and *Sargassum vulgare*) [189,190].

### 4.5. Terpenoids and Sterols

**Figure 6:**
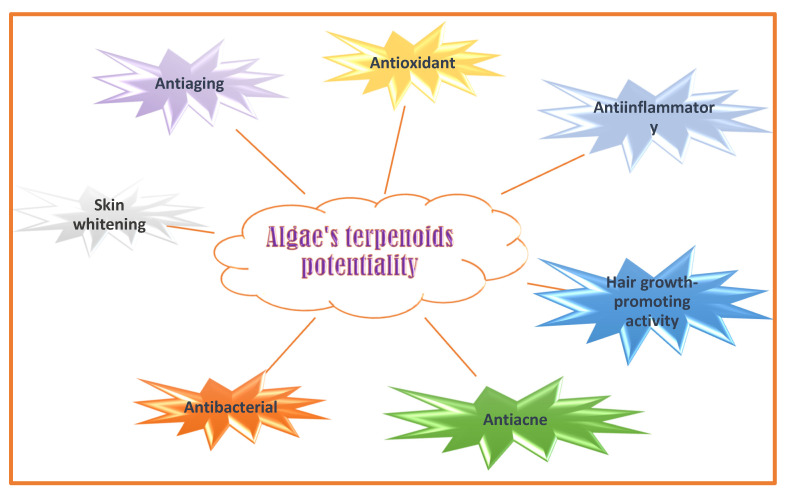
Algaes’ terpenoids potentialities in nutricosmetic formulation.

Mono- di- tri-and sesquiterpenoids were isolated from macro- and microalgae. Isoprenoid C5-subunits’ condensation forms terpenoids [191]. Terpenoids can act as antioxidants, antiaging (improving antioxidant enzymes such as catalase, superoxide dismutase, and glutathione peroxidase levels) [192], anti-inflammatory, skin-whitening (by inhibition of tyrosinase activity) [192], antibacterial (against gram-negative and gram-positive bacteria) [193], and anti-acne bioactive molecules (acting against *Staphylococcus aureus*, a gram-positive bacterium associated with acne vulgaris pathology) (Figure 6) [194,195].

The tetraprenyltoluquinol meroterpenoid from *Sargassum muticum *and meroterpenoid have antioxidant and anti-photoaging properties [196].

Loliolide monoterpenoid abundant in brown algae *(Sargassum crassifolium* and *Padina tetrastromatica*), and red algae (*Corallina pilulifera*), improve hair growth via AKT-mediated WNT(wingless-int) signaling activation [197].

The brown algae meroterpenoids determine skin-whitening [198]. The sesquiterpene 5β-Hydroxypalisadin B [(2*R*,5*R*,7*S*,9a*S*)-7-bromo-2-(bromomethyl)-3,6,6,9a-tetramethyl-2,5,5a,6,7,8,9,9a-octahydrobenzo[b]oxepin-5-ol] isolated from the red algae *Laurencia snackeyi* (Weber Bosse), and diterpenoid methyl 16(13→14)-abeo-7-labdebe(12-oxo)carboxylate from the red algae *G. salicornia*, have anti-inflammatory properties [199,200].

Phenolic terpenoids (Figure 5) are mero diterpenoids (chromanols, chromenes, plastoquinones) found in red and brown seaweeds [201]. Chromene-based molecule isolated from *Gracilaria opuntia* has shown antiinflammatory and antioxidant activity [202]. Tetraprenyltoluquinol meroterpenoids isolated from *Halidrys siliquosa* have shown antibacterial activity against *Cobetia marina* (ATTC 25374), *Marinobacterium stanieri* (ATCC 27130), *Vibrio fischeri* (ATCC 7744), and *Pseudoalteromonas haloplanktis* (ATCC 14393) [203,204].

Finally, seaweed contains sterols. Sterols are similar to cholesterol but have an alkyl substituent at C-24. In algae, they are present in free form or conjugated with fatty acids (e.g., oleate) or sugars (e.g., glucose) [205]. The brown algae contain principally fucosterol, red algae cholesterol, and green algae, a mixture of ergosterol, 28-isofucosterol, β-sitosterol, cholesterol, and poriferasterol [205]. Sterols can regulate membranes’ permeability and fluidity and have antioxidant, anti-inflammatory, and antiphotodamage [206,207,208,209].

### 4.6. Pigments

The algaes’ pigments can be brown (carotenes and xanthophylls), green (chlorophylls), and red (phycobilins).

Carotenoids are lipophilic isoprenoid molecules that can be used as natural color enhancers in food, cosmetic, and pharmaceutical formulations. They comprise carotenes and xanthophylls (e.g., β-carotene, zeaxanthin, astaxanthin, and fucoxanthin) with photoprotective, antioxidant, and antiaging properties [210]. β-Carotene acts as provitamin A and has antioxidant, anti-inflammatory, and antiaging properties [211,212]. Astaxanthin (xanthophyll compound) and fucoxanthin (in brown algae) have antioxidant and anti-macular degeneration properties [213,214,215]. Moreover, fucoxanthin can improve the fat-burning rate in adipose tissue [216]. The zeaxanthin (in red and green macroalgae) has whitening properties being able to control the tyrosinase’s activity (enzyme able to produce melanin) [217].

Chlorophylls are characterized for containing a porphyrin ring with a central magnesium ion. They protect algae against oxidative stress due to UV radiation [218].

Chlorophyll derivatives (pheophytin, pyropheophytin, and pheo-phorbide) also have antioxidant and antimutagenic abilities [83]. The chlorophyll level in the macroalgae is improved by overexposure to UV radiation [210].

Phycobiliproteins are mainly present in macroalgae and red macroalgae. They have antioxidant, antiaging, anti-inflammatory, and immune-modulator activities [218]. The phycobiliproteins remain stable in pH ranges between 5 and 9, allowing their use in cosmetics (e.g., eye shadows, creams, makeup, and lipsticks) [219].

The phycocyanin *R*-phycoerythrin and allophycocyanin are employed as colorants in cosmetic formulations [220].

## 5. Technological Properties of the Algae Metabolites

**Figure 7:**
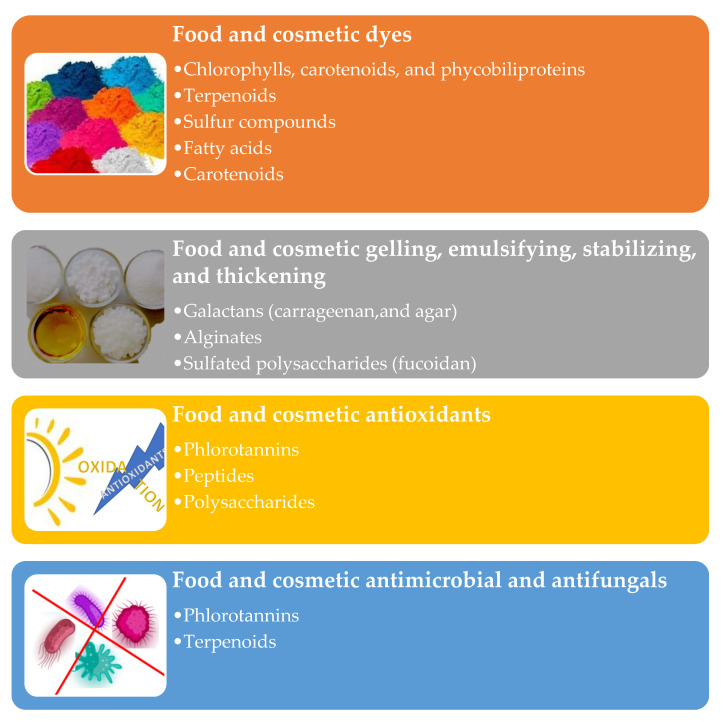
Algae metabolites’ technological potentialities.

Algae metabolites can be used as technical ingredients to enhance cosmetics’ color, texture, and stability (Figure 7) [114,220]. Their efficiency and stability can be improved with carriers (e.g., nano/microparticles, liposomes, hydrogels, and emulsions) [221,222,223,224,225]. The cosmetic industry uses principally synthetic or mineral dyes, some of which can cause allergies. Algae pigments (e.g., chlorophylls, carotenoids, and phycobiliproteins) may be a valid alternative [226,227]. The FDA has authorized spirulina extracts (containing phycobiliproteins) as colorants in human foods [228,229].

Algae terpenoids, sulfur compounds, fatty acids, and carotenoids can be employed as flavoring in cosmetic, food, and nutraceutical formulations [230,231].

Algae polysaccharides can be employed for their rheological behavior. Carrageenan, agar, and alginate can be used for gelling, emulsifying, stabilizing, and thickening since they form highly viscous solutions in water [232,233].

They are GRAS substances considered safe for human consumption by the European Food Safety Authority and the Food and Drug Administration [234]. The fucoidan (from *U. pinnatifida* and *F. vesiculosus*) was authorized by the European Commission (Regulation 2017/2470) in foods and food supplements [235]. The algal phlorotannins, peptides, and polysaccharides can protect the nutricosmetic formulation’s lipidic component from oxidative deterioration and maintain their original sensorial properties [236,237,238]. Finally, algae’s terpenoids and phlorotannins can be employed as preservative agents against bacteria and fungi [239].

## 6. Cosmetic Potenziality of Algae Metabolites

**Figure 8:**
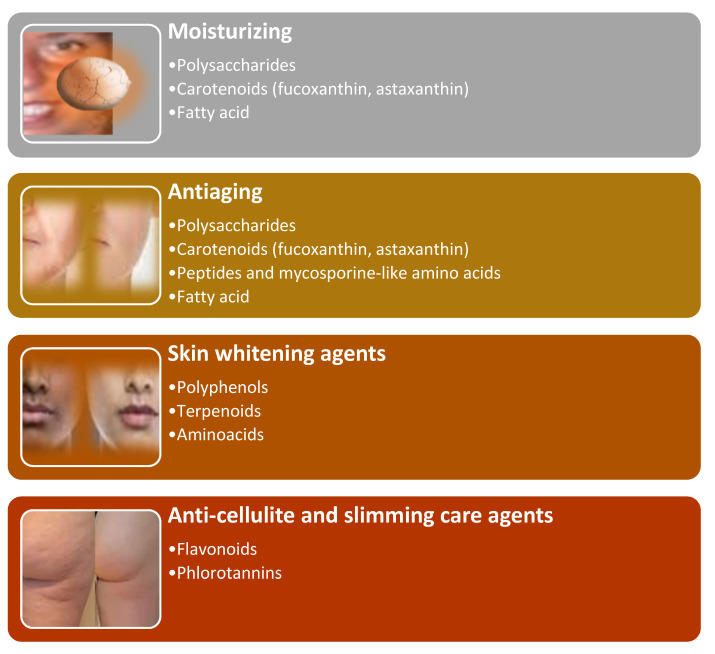
Algae metabolites’ nutricosmetic potentialities.

Algae’s metabolites in nutricosmetic products can be used as moisturizing, antiaging, skin whitening, anti-cellulite, and slimming care agents (Figure 8).

### 6.1. Algae Metabolites in Moisturizing Formulations

The skin protects the body from the environment by maintaining an efficient epidermal barrier against injuries and preventing excessive water loss. The Natural Moisturizing Factors (NMF) present in the *Stratum corneum*, the epidermis’ outermost layer, contain lactic acid, pyrrolidone carboxylic acid urea, and amino acids (e.g., serine) able to uptake water [240]. The fat metabolism (in sebaceous glands) and conversion of phospholipids to free fatty acids produce glycerol [241] transported by the aquaporins through the epidermis via specific water/glycerol channels. Aquaporin expression is stimulated by retinoic acid [242].

Cosmetic products for dehydrated skin are based on ingredients with film-forming and occlusive properties (e.g., vegetable oils, fatty alcohols, hydrocarbons, waxes, silicones, and butter, etc.), or humectant agents, (which improve the Stratum corneum ability to capture water, e.g., glycerin or propylene glycol) [243] or moisturizers that penetrate the corneous layer permitting water to be retained [244].

The algae’s polysaccharides (mainly made by green and brown algae), oligosaccharides, and fatty acids can be employed as moisturizing agents. The polysaccharides (mainly marine green algae) moisturize slower and retain more moisture than glycerin [245]. A moisturizing retention rate of over 94% was referred to the polysaccharides belonging to brown algae (e.g., *Sargassum horneri* [246], *Sargassum vachellianum* [247], *Sargassum hemiphyllum* [248]. When applied topically, the sulfated polysaccharides (from red algae Porphyra haitanensis) enhance dry facial skin features and moisturization, regulating the keratinized envelope’s maturation of the stratum corneum and dermal-epidermal junction [245]. Low molecular weight and sulfated group enhance the moisture-retention and absorption abilities [192]. The alginates (extracted from brown macroalgae) and agar (from red macroalgae) have hydrating properties linked to their ability to conserve water [249].

The lipids can maintain skin integrity and purity, restoring barrier permeability and preventing skin dehydration due to unsaturated fatty acid deficiency in the skin. The brown macroalgae *Laminaria ochroleuca* produces numerous unsaturated fatty acids (e.g., oleic acid, linoleic acid, linolenic acid, and palmitoleic acid) with moisturizing properties widely used in oil/water emulsions to maintain water loss in the skin [250]. Oral or topical administration of astaxanthin (carotenoid) can improve skin moisture by improving the aquaporin levels (substances that regulate skin moisture and function) [251]. The green microalga *Cladophora glomerata* contains unsaturated fatty acids C16:1 (n-7) and C18:1 (n-3) and saturated fatty acids (palmitic acid C16:0) that can be used as emollients and to reduce water loss, and sulfated polysaccharides that have moisturizing properties [252].

### 6.2. Algae Metabolites in Antiaging Formulations

During the aging process, the dermis change. The matrix metalloproteinases (MMPs) activity increases, and collagen (one of the significant components of the extracellular matrix) levels decline [253]. Intrinsic (natural skin degradation) and extrinsic (ROS generated by UV radiation, pollution, etc.) factors can cause dryness, thinning, laxity, enlarged pores, fragility, wrinkles, and fine lines. The bioactive molecules that inhibit metalloproteinases help constrain aging. Sulfated polysaccharides (found in *Phaeophyceae*, *Rhodophyceae*, and *Chlorophyceae*), and polyphenols, derived from phloroglucinol, downregulate the metalloproteinases activity [254,255]. Fucoidan can regulate fibroblasts and restore skin tissue function [256]. Carrageenans act as thickening, water-binding [257] antioxidant, and antiphotoaging bioactive molecules [258]. Galactan of *P. haitanensis* decreases the cell’s aging process regulating the p53-p21 signaling pathway [259]. Astaxanthin (a carotenoid) protect against photo-oxidation [215]. Fucoxanthin upregulates the fibroblasts’ procollagen synthesis and decreases the expression of matrix metalloproteinases in wrinkle care cosmetics [260]. Amino acids and peptides from macroalgae stimulate collagen production in the skin [219]. Mycosporine-like amino acids act as antioxidants, antiinflammatories, UV-absorbing agents, and down-regulate the protein-glycation and collagenase activity [126]. Ascorbyl palmitate antioxidant effect is used in anti-aging and anti-wrinkle formulations [261,262].

### 6.3. Algae Metabolites in Skin Whitening Formulations

The pigmentation process controls the color of mammalians’ hair, skin, and eyes [263].

Tyrosinase enzyme regulates the conversion of l-tyrosine and l-3,4-dihydroxyphenylalanine (L-DOPA) in pheomelanin (red-orange pigment) and eumelanins (dark brown pigments) [264,265]. When tyrosinase is upregulated, hyperpigmentation determines freckles, age spots, irregular dark patches, and nevi. On the contrary, when tyrosinase is downregulated, melanin synthesis is reduced, and white patches (e.g., vitiligo) are observed [266]. Some algae’s phenols, terpenoids, amino acids, sugars, and amines, used as skin-whitening agents, are tyrosinase inhibitors [192,267]. Red algae, the richest sources of mycosporine-like amino acids, are a helpful source of whitening bioactive molecules for the cosmeceutical industry [268].

### 6.4. Algae Metabolites in Anticellulite and Slimming Care Formulations

In cosmetology, the term “slimming product” is preferred to “anti-cellulite” since cellulite is a disorder produced by a deep dermis and subcutaneous tissue change and, therefore, a term linked to the medical world [269]. Cellulite has a multifactorial etiology [270]. Estrogens and microcirculation disorders (decreasing blood flow in the capillaries), the nervous system (downregulating the lipolysis process), and genetic factors can be involved. The slimming product objectives include correcting the” orange peel” appearance and “mattress symptom” characterized by roughness, skin surface collapse, and yellow-gray skin tone.

The iodine-rich algae (e.g., *Laminaria Japonica*) can be used to constrain cellulite since iodine regulates the thyroid hormones’ synthesis, which boosts lipolysis by facilitating the penetration of fatty acids into the mitochondria [192,271,272].

Examples of patents claiming the use of algae and algae metabolites in cosmetic formulations are reported in Table 1.

## 7. Macroalgae Biomass in a Circular Economy Perspective

Recent studies have considered algae a sustainable and environmentally friendly way to eliminate contamination from wastewater since they use low energy and pollutants to grow [282] and to produce biomass [283]. The dry biomass or wet paste of microalgae can be employed to extract bioactive metabolites. Selling prices improve from biomass to secondary metabolites [284]. The “chemicals and materials” and bio-energy market use whole biomass. The “food, pharmaceuticals and personal care” markets employ primary and secondary metabolites in the feed, food, supplement, nutraceutical, and cosmeceutical preparations. Raw biomass can enhance the soil organic matter and water capacity in agriculture. The defatted biomass from biodiesel extraction, mixed with water, can produce biogas after anaerobic digestion and can be used to extract metabolites. For example, the residual lipids can be upcycled as supplements in animal feed [285]. Glycerol, a byproduct of the microalgal lipids’ transesterification to biodiesel, can be converted to solvents, polymers, and aliphatic polyesters, to generate electricity directly in biofuels cells or to prepare foods, cosmetics, and drugs [286]. The digestate resulting from biogas production can be employed as fertilizer and conditioner. Microalgae biomass can be employed as a food supplement, feed additive, and feed in the aquaculture of crustaceans, fishes, and mollusks [287]. Proteins, lipids (e.g., phospholipids and glycolipids), starches, and sugars can be used in food, nutraceutical and personal care, and drug products. Chlorophylls and carotenoids can be used as food and cosmetic dyes [288]. Sterols can be used as anti-inflammatory and cholesterol-lowering bioactive molecules in foods and supplements [289]. PUFA and oxylipins can be used as nutricosmetics, food supplements, and feeds [290,291]. The cost, microbial and chemical contaminants’ accumulation, and the lack of technology viable for large-scale applications give a setback to algal wastewater treatments [292]. Different is the speech of the potential use of the beach-cast macroalgae. Tonnes of marine algae are removed per year and dumped in landfills. Very few registers of abundance and composition of beach-cast marine algae worldwide exist. These algae should be less rich in toxic products than algal wastewater and probably do not need detoxification processes [293]. Thus, it would be enough to imagine strategies for large-scale extraction of bioactive molecules to take advantage of this natural and eco-sustainable source of raw materials for industry.

## 8. Conclusions

Algae are rich sources of bioactive molecules (amino acids, carbohydrates, lipids, phenols, and terpenoids), helpful for improving the functional, stability, and sensorial characteristics of nutricosmetic products. The vast array of bioactive molecules makes algae an attractive and versatile resource to obtain safe bio-based products. Algae extract and their purified metabolites are gaining increasing commercial importance. Many patents concerning algae extracts or metabolites application in nutricosmetic products have been registered recently. Unfortunately, many do not report the mechanisms responsible for cosmetic performance. It would be helpful that more works evaluate the algae extract profiles to identify functional properties, stability, compatibility, and toxicology aspects to facilitate the development of new nutricosmetic. Concerning the use of algae to eliminate pollution from wastewater and produce biomass from which obtain bioactive molecules, the cost, non-sterile conditions, and lack of technology viable for large-scale applications limit their application. Better potential can be seen for the recycling of beach-cast macroalgae.

## Figures and Tables

**Table 1:** Examples of algaes’ use in the cosmetic field.

Patent No.	Title	Reference
**PATENTS CONCERNING THE IMPROVED SKIN APPEARANCE**		
US20210093540A1	Seaweed-derived cosmetic compositions	[273]
US10493007B2	Microalgae-derived compositions for improving the health and appearance of skin	[274]
**PATENTS CONCERNING ANTIAGING EFFECTS**		
US20210161980A1	Seaweed extracts, isolated compounds, and methods of treatment	[275]
US9717932B2	Marine extracts and biofermentations for use in cosmetics	[276]
CN105777933A	Preparation of algal polysaccharides and application of algal polysaccharides in cosmetics	[277]
TW200914061A	Method for using green algae extract to retard aging of skin cells and cosmetic composition containing green algae extract	[278]
**PATENTS CONCERNING ANTIWRINKLE EFFECTS**		
PCT/KR2011/008910	Cosmetic composition containing gulfweed extract sea staghorn extract and brown seaweed extract.	[279]
**PATENTS CONCERNING ANTI-WHITENING EFFECTS**		
WO2012011907A1	*Laminaria Saccharina* extract and vitamin B3 as whitening agents. WIPO (PCT)	[280]
**PATENTS CONCERNING HAIR EFFECTS**		
EP1433463B1	Use of algal proteins in cosmetics.	[281]

## Data Availability

Not applicable.
